# 

**Published:** 1891-02

**Authors:** 


NOTICE.

***All articles for publication, books for review, business communications, etc., must be sent to Editors, 1125 Spruce Street, Philadelphia.***

***Subscribers will please notice that this Journal will be sent until arrears are paid and it is ordered discontinued.***

